# Synergetic Effect of Dy_2_O_3_ and Ca Co-Dopants towards Enhanced Coercivity of Rare Earth Abundant RE-Fe-B Magnets

**DOI:** 10.1186/s11671-017-2407-z

**Published:** 2017-12-13

**Authors:** Yingfei Li, Na Tian, Xiaodong Fan, Caiyin You, Wenli Pei, Zhenxiang Cheng

**Affiliations:** 1 School of Materials Science & Engineering, Xi’an University of Technology, Xi’an, 710048 People’s Republic of China; 2 Institute for Superconducting & Electronic Materials, University of Wollongong, Wollongong, NSW2500 Australia; 3 Key Laboratory for Anisotropy & Texture of Materials, Northeastern University, Shenyang, 110819 People’s Republic of China

**Keywords:** Abundant RE-Fe-B magnets, Coercivity, Dy_2_O_3_ and ca co-dopants, Synergetic effect

## Abstract

Low coercivity is the main disadvantage of RE-Fe-B permanent magnets containing highly abundant rare earths (RE: La, Ce) from the application point of view, even though they exhibit many cost and resource advantages. In this work, an industrial mixed rare earth alloy (RE_100_ = La_30.6_Ce_50.2_Pr_6.4_Nd_12.8_) with a high amount of the more abundant elements was adopted to fabricate RE-Fe-B permanent magnets by means of mechanical alloying accompanied by post-annealing. A synergetic effect towards enhancing the coercivity was observed after co-doping with Dy_2_O_3_ and Ca, with the coercivity increasing from 2.44 kOe to 11.43 kOe for co-dopant percentages of 7 wt.% Dy_2_O_3_ + 2.3 wt.% Ca. Through analysis of the phase constituents and microstructure, it was determined that part of the Dy atoms entered the matrix of RE_2_Fe_14_B phase to enhance the magnetocrystalline anisotropy; due to the reductive effect of Ca on Dy_2_O_3_, nanocrystals of Dy-rich RE_2_Fe_14_B were present throughout the matrix, which could increase the resistance to domain wall movement. These are the dominant factors behind the improvement of the coercivity of the RE-Fe-B magnets with highly abundant RE elements.

## Background

Highly abundant rare earth elements, such as La and Ce, have been used to fabricate rare earth permanent magnets for the purposes of reducing costs and conserving usage of rare earth resources [1–5]. Nevertheless, the permanent magnets with high concentrations of La and Ce exhibit significantly degraded performance because the magnetocrystalline anisotropy of the 2:14:1 phases La_2_Fe_14_B and Ce_2_Fe_14_B is much lower than for their Nd_2_Fe_14_B counterpart [6]. So far, most work has been focused on the substitution of La and Ce for Nd in Nd-Fe-B-based magnets [7–14]. The performance of these permanent magnets can be much enhanced through adjusting the microstructure. In addition, it has been widely reported that doping with heavy rare earth elements (Dy or Tb) is a highly useful way to improve the magnetic performance [15, 16], for example, by enhancing the coercivity and thermal stability. It was reported that both the coercivity and the thermal stability of Nd_2_Fe_14_B-type magnets can be enhanced through doping with Dy_70_Cu_30_ [17, 18] or Dy_80_Al_20_ [19]. The increase in the coercivity was 4.4 kOe and 9.0 kOe for the 2 wt.% Dy_70_Cu_30_ [18] and 4 wt.% Dy_80_Al_20_ [19] samples, respectively. As is well known, these heavy rare earth alloys are much more expensive. Thus, the cost advantages of La-Ce-Fe-B-based permanent magnets could be diminished if pure heavy rare earth metals or alloys were selected as dopants. Therefore, it would be worthwhile to find a route to match the enhancing effects of the pure heavy rare earth metals or alloys by using low-price compounds of heavy rare earth elements (Dy or Tb), for example, in the form of oxides. In fact, the addition of oxides could be helpful for improving the high frequency behavior of the magnets due to their high resistivity.

Recently, the reduction-diffusion process through Ca reduction of the rare earth oxides has been widely investigated to fabricate high-performance rare earth permanent magnets, such as Nd_2_Fe_14_B- and Sm_2_Fe_17_N_*x*_ *-*based magnets [20, 21]. In this work, a cheap industrial rare earth alloy (RE_100_ = La_30.6_Ce_50.2_Pr_6.4_Nd_12.8_) with a high amount of the abundant elements was adopted as the source material. Dy_2_O_3_ was utilized as the precursor of the heavy rare earth element Dy to improve the magnetic performance rather than the expensive pure heavy rare earths or their metallic alloys [15–19]. Furthermore, Ca was also co-doped to promote the beneficial effects of Dy_2_O_3_ through the reducing reaction between Dy_2_O_3_ and Ca. A coercivity as high as 11.43 kOe was achieved for the magnets with a concentration of the abundant rare earth elements La and Ce that was higher than 80 at.%. This work suggests a facile way of making use of the Ca-reducing effect to strengthen the enhancement of the magnetic properties of rare earth permanent magnets by the use of rare earth oxides.

## Methods

An industrial rare earth (RE) alloy with abundant La and Ce (RE_100_ = La_30.6_Ce_50.2_Pr_6.4_Nd_12.8_, 99.5 wt.%, denoted as RE in this work), iron (99.9 wt.%), and iron-boron alloy (99.5 wt.%) with the nominal composition of RE_13.6_Fe_78.4_B_8_ were arc melted. The melted alloy was smashed into powder. In a high-purity argon-filled glove box, the powders were sealed in a hardened steel vial containing steel balls 12 mm diameter, with the powder-ball mass ratio of 1:16. Dy_2_O_3_ and Ca powders with a particle size of about 100 μm were added. Ball milling was performed using a high-energy ball mill with a rotation speed of 700 rpm for 5 h. In order to investigate the effects of the Dy_2_O_3_ and Ca dopants on the magnetic properties, 2.3 wt.% Ca (sample denoted as MC), 3 wt.% Dy_2_O_3_ (sample denoted as M3D), 7 wt.% Dy_2_O_3_ (sample denoted as M7D), and the co-dopants 2.3 wt.% Ca and 7 wt.% Dy_2_O_3_ (sample denoted as M7 DC) were, respectively, added before milling. The pure RE-Fe-B sample without the dopant was denoted as RM. Subsequently, the milled powders were annealed at 620–780 °C for 10 min in a vacuum environment (better than 1.3 × 10^−3^ Pa). The phase components were analyzed with an MSAL-XD2 mode X-ray diffraction instrument (Cu-Kα, *λ* = 0.15406 nm). Hysteresis loops were measured using a LakeShore 7404 Model vibrating sample magnetometer (VSM) at room temperature, for which the sample powder was solidified into a cylinder 2 mm in diameter and 4 mm in length with epoxy resin, and the results were corrected by using an experimentally determined demagnetization factor of 0.28 [22]. Magnetic performances at low and high temperature were characterized by a Quantum Design Versa-lab and DynaCool physical properties measurement system (PPMS). A JEM-2100F transmission electron microscope (TEM) was used to carry out the microstructural observations.

## Results and Discussion

**Fig. 1:**
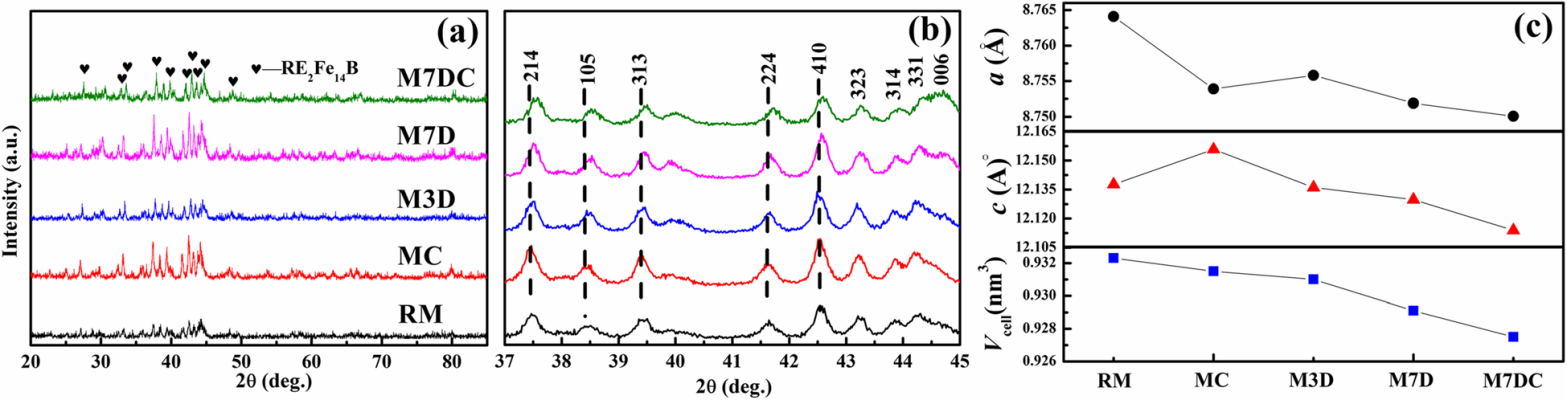
**a** XRD patterns of samples annealed at 700 °C; **b** enlarged XRD patterns with slow scanning from 37° to 45°; **c** lattice parameters *a*, *c*, and cell volume for the samples

The samples annealed at 700 °C were selected to characterize the phase constituents. Figure 1 presents the X-ray diffraction (XRD) patterns of the annealed samples. All the samples mainly consisted of RE_2_Fe_14_B matrix phases [5, 6]. Slow scanning from 37° to 45° was performed to study the lattice variations after doping with Dy_2_O_3_ and Ca, as shown in Fig. 1b. The lattice parameters, *a* and *c*, and the cell volume (Fig. 1c) were evaluated by Jade software in terms of the XRD patterns of Fig. 1b. The results indicated that the Ca, as a single dopant, caused obvious shrinkage of the 2:14:1 phase crystal cell, indicating the substitution of Ca for the rare earth elements, since the Ca metallic radius is much larger than the value for Fe [23]. The Dy_2_O_3_ dopant caused shrinkage of the crystal cells, too, suggesting Dy entrance into the 2:14:1 phase. With increasing Dy_2_O_3_ content, the cell shrinkage became serious, presenting lower values of the lattice parameters. Regarding the sample with Dy_2_O_3_ and Ca as co-dopants, the total volume shrinkage of about 0.0048 (nm^3^) was above the sum of the values for 2 wt% Ca (0.0008 nm^3^) and 7 wt.% Dy_2_O_3_ (0.0032 nm^3^) as single dopants, implying that the Ca promoted the shrinkage due to more Dy entrance into 2:14:1 phase.

**Fig. 2:**
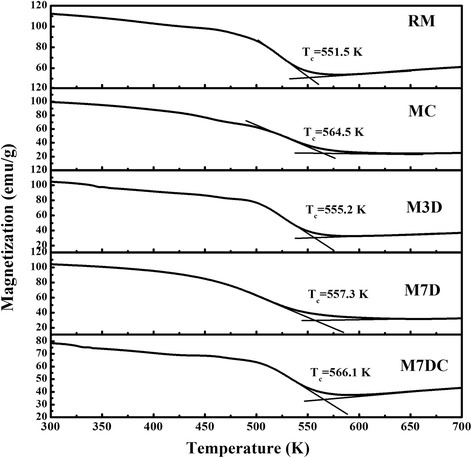
Magnetization variation of the samples with temperature from 300 K to 700 K

The thermal magnetic behavior of the samples was investigated to further clarify the dopant occupation in the 2:14:1 matrix phase. Figure 2 shows the variation in magnetization of samples annealed at 700 °C as a function of temperature from 300 to 700 K, in which the magnetic field of 2 T was applied to saturate the magnetic moment. On heating the samples, the ferromagnetic-paramagnetic phase transition of 2:14:1 phase took place at the Curie temperature (*T*_*C*_). As indicated in Fig. 2, *T*_*C*_ was slightly increased from 551.5 to 557.3 K after doping with Dy_2_O_3_, but it exhibited a significant increase from 551.5 to 564.5 K with Ca dopant. There is a slight further increase in *T*_*C*_ from 564.5 to 566.1 K after co-doping with Ca and Dy_2_O_3_. These features are consistent with the XRD results, indicating the entrance of Dy or Ca into the lattice of 2:14:1 phase. It was also observed that the temperature of the spin re-orientation varied consistently with the dopants (data not shown here).

**Fig. 3:**
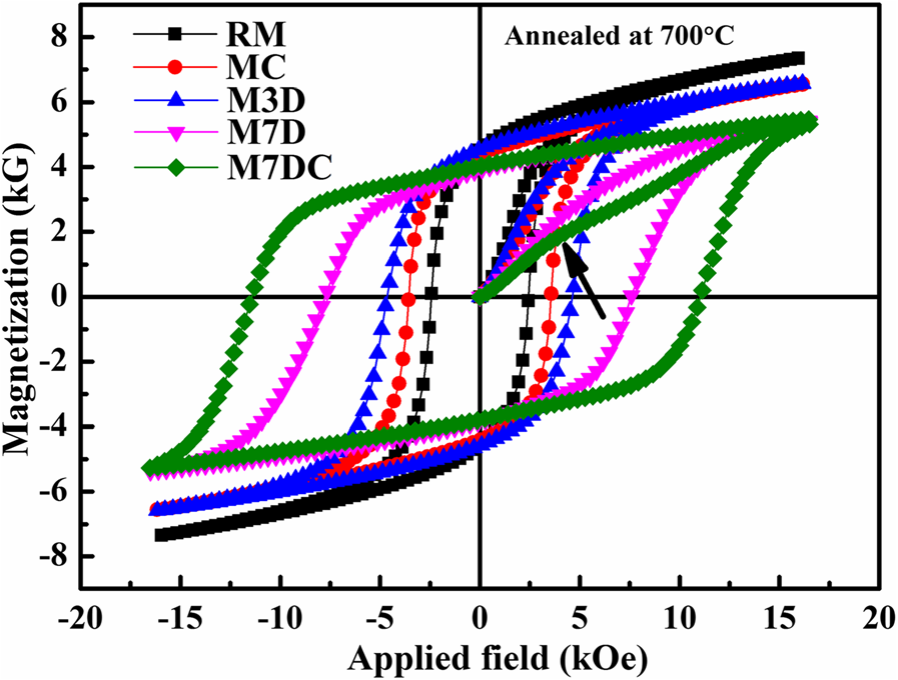
Room temperature magnetic performance of the samples annealed at 700 °C. The black arrow indicates a region of strong domain pinning

**Fig. 4:**
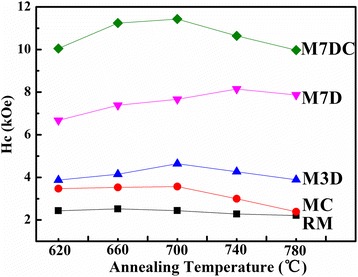
Coercivity of the samples as a function of the annealing temperature

Figure 3 presents typical magnetic hysteresis loops of the samples annealed at 700 °C. The coercivity increased, and the saturation magnetization decreased in the presence of the dopants. The dependence of the coercivity on the annealing temperature is shown in Fig. 4. With Ca doping, the coercivity of all the samples was slightly enhanced. Dy_2_O_3_ dopant was also helpful for improving the coercivity. On doping with 7 wt.% Dy_2_O_3_, the coercivity increased from 2.44 to 7.65 kOe when the sample was annealed at 700 °C. Although the 2.3 wt.% Ca as a single dopant did not contribute a large enhancement of coercivity (about 1.2 kOe), Dy_2_O_3_ and Ca as co-dopants caused more significant enhancement of coercivity (about 9.1 kOe) than the total effect of each individual dopant (about 6.3 kOe), as shown in Fig. 4.

**Fig. 5:**
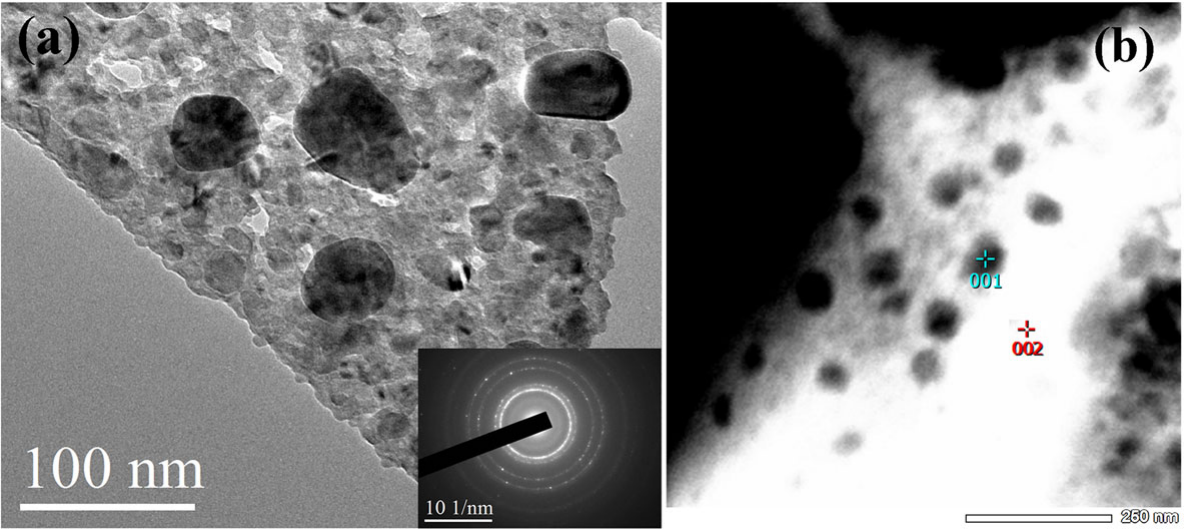
**a** Bright field TEM image of the RE_13.6_Fe_78.4_B_8_ with 7 wt.%Dy_2_O_3_ and 2.3 wt.%Ca co-dopants (inset: selected area diffraction pattern); **b** STEM image showing the dark coarse grains

**Fig. 6:**
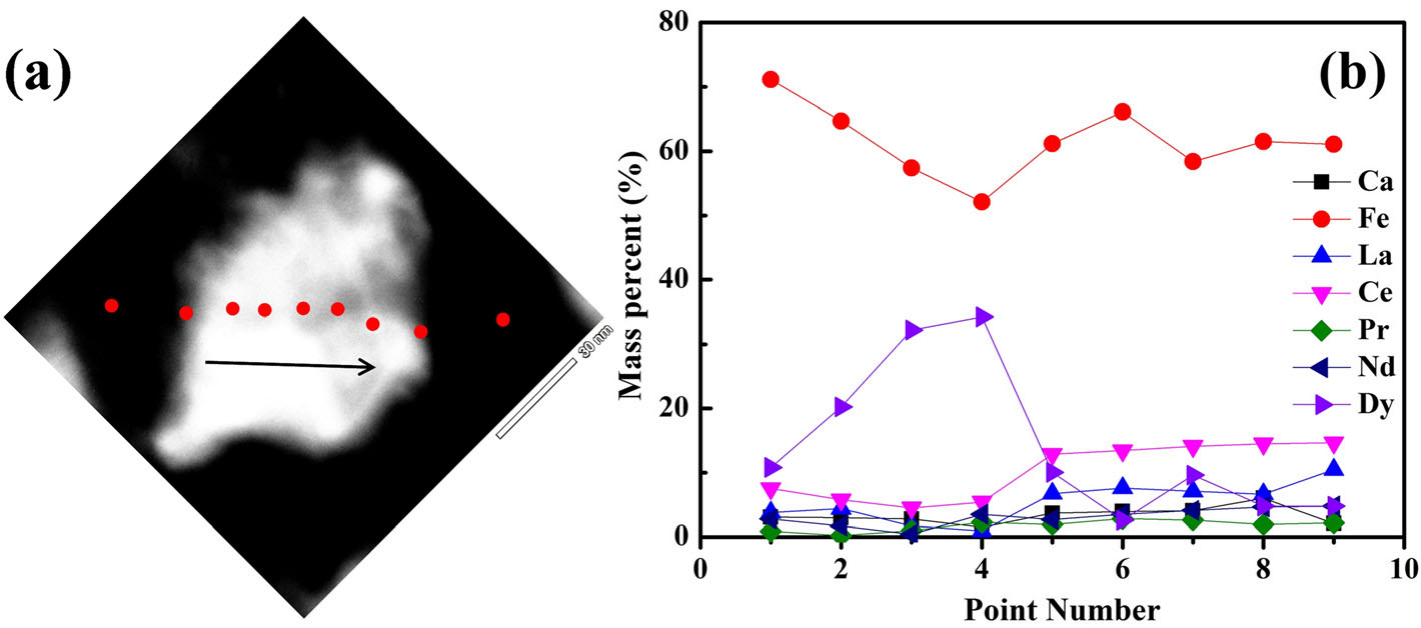
**a** Point detection by EDS on RE_13.6_Fe_78.4_B_8_ sample co-doped with 7 wt.%Dy_2_O_3_ and 2.3 wt.%Ca, and **b** elemental concentration of each detected site

The co-doped sample annealed at 700 °C, which presented the highest coercivity, was selected for the microstructural observations, as shown in Figs. 5 and 6. Figure 5a presents a bright field TEM image, which exhibits a nanocrystalline structure (inset: corresponding selected area diffraction pattern). In addition, there are some coarse grains embedded within the matrix. Scanning TEM (STEM) mode was selected to detect the chemical information. Figure 5 presents the STEM image, in which dark coarse grains appear, dotting the sample. Through energy dispersive spectroscopy (EDS) analyses, it could be shown that the dark coarse grains contain high fractions of Dy and Ca compared with the other regions, as listed in Table 1. Note that the contents of oxygen and boron are not included in Table 1 because there is less EDS accuracy for the light elements. A further characterization of the elemental chemistry was carried out by point detection in EDS along one coarse grain, as shown in Fig. 6. Figure 6b presents the elemental concentrations at each detected site. It is clear that there is a Dy-rich region containing less Ce and La.

**Table 1:** EDS results from points 001 and 002 in Fig. 5b of RE_13.6_Fe_78.4_B_8_ with 7wt.%Dy_2_O_3_ and 2.3wt.%Ca co-dopants

Element (at.%)	Ca	Fe	La	Ce	Pr	Nd	Dy
Point 001	12.47	72.70	3.40	5.96	1.06	1.85	2.56
Point 002	7.32	77.68	3.73	6.92	0.81	2.12	1.42

Note that the concentrations of oxygen and boron are not included because there is less EDS accuracy for the light elements

As shown in Fig. 5, the coercivity can be enhanced by doping with Ca or Dy_2_O_3_. The co-dopants Ca and Dy_2_O_3_ give a strong improvement in comparison to each single dopant alone. It can be seen that the initial magnetic curve of the co-doped sample exhibits a mixed mechanism of nucleation and domain wall pinning, as indicated by the arrow in Fig. 3. When the applied field is under 5 kOe, the initial magnetic curve of the co-doped sample presents the features of the nucleation mode; after the external field is higher than 5 kOe, the reversal of magnetic domains becomes difficult, showing the feature of domain wall pinning. In terms of the microstructural observations, there were some coarse grains with a high concentration of Dy element (Figs. 5 and 6), which could act as pinning sites due to the high magnetocrystalline anisotropy.

XRD analysis shows that doping with Ca shrank the *a*-axis parameter and expanded the *c*-axis parameter, while doping with Dy_2_O_3_ shrank both the *a* and the *c* axis parameters (Fig. 1c). Shrinkage of both the *a* and the *c* axis parameters occurred for the sample with the co-dopants. The Pearson’s metallic radius of Dy (0.1773 nm) is smaller than for La (0.1877 nm), Nd (0.1821 nm), and Pr (0.1828 nm) [23]. Thus, shrinkage of the unit cell of RE_2_Fe_14_B takes place with increasing amounts of Dy. The Ca prefers to replace the RE atoms due to its large metallic radius (0.1773 nm) [23], causing the expansion of the *c*-axis parameter. Nevertheless, the cell volume of RE_2_Fe_14_B was reduced due to the shrinkage of the *a*-axis parameter after doping with Ca. In contrast to the sample with 7 wt.% Dy_2_O_3_, the shrinkage of both *a* and *c* appeared after additional doping with Ca, rather than the shrinkage of the *a* parameter alone, as in the case of Ca single doping.

As reported previously, the high energy mechanical milling caused a partially amorphous alloy, and re-crystallization behavior took place in the milled alloys during the post-annealing at relatively low temperature [22]. According to the Standard Electrode Potentials [24], Ca (−2.868 V) has a lower potential than the rare earth elements involved in this work, while Dy (−2.295 V) has the highest potential among the rare earth elements. Making use of the chemical potential differentials, a reduction-diffusion process between Ca and the rare earth oxides took place in the fabrication of the rare earth permanent magnets [20, 21]. Thus, a reductive reaction would occur between the Ca and Dy_2_O_3_ during the mechanical milling and post-annealing. The reduced Dy atoms can take part in the recrystallization of RE_2_Fe_14_B phase, suggesting that the Ca could enhance the entrance of Dy into the 2:14:1 matrix rather than its own entrance. In addition, this local reductive reaction could promote elemental diffusion and mobility, resulting in the formation of some coarse grains, as shown in Figs. 5 and 6, which contain high amount of Ca and Dy. Therefore, the coercivity was significantly enhanced for the co-dopants due to the significant increase in magnetocrystalline anisotropy originating from more Dy in the 2:14:1 phase. A better magnetic performance could also be expected if the trace CaO could be removed.

## Conclusions

The coercivity of a RE_2_Fe_14_B-based permanent magnet, with the RE content coming from an industrial mixed alloy of highly abundant rare earth elements (RE_100_ = La_30.6_Ce_50.2_Pr_6.4_Nd_12.8_), was significantly enhanced from 2.44 kOe to 11.43 kOe through doping with Dy_2_O_3_ and Ca. Based on the variations in the lattice parameters, it could be deduced that Ca promotes the entrance of Dy into 2:14:1 phase due to its reducing effect on Dy_2_O_3_. This work proposes a way to fabricate high-coercivity permanent magnets with a high concentration of highly abundant rare earth elements.
